# Boosted Spontaneous Formation of High‐Aspect Ratio Nanopeaks on Ultrafast Laser‐Irradiated Ni Surface

**DOI:** 10.1002/advs.202200761

**Published:** 2022-05-26

**Authors:** Anthony Nakhoul, Anton Rudenko, Claire Maurice, Stéphanie Reynaud, Florence Garrelie, Florent Pigeon, Jean‐Philippe Colombier

**Affiliations:** ^1^ Univ Lyon UJM‐Saint‐Etienne, CNRS, IOGS, Laboratoire Hubert Curien, UMR5516 St‐Etienne 42023 France; ^2^ Univ Lyon Mines Saint‐Etienne, CNRS, Centre SMS, Laboratoire Georges Friedel, UMR5307 St‐Etienne 42023 France; ^3^ Arizona Center for Mathematical Sciences and College of Optical Sciences University of Arizona Tucson AZ 85721 USA

**Keywords:** high aspect‐ratio, hydrodynamic simulation, nanoengineering, nanopeaks, ultrafast laser

## Abstract

The capacity to synthesize and design highly intricated nanoscale objects of different sizes, surfaces, and shapes dramatically conditions the development of multifunctional nanomaterials. Ultrafast laser technology holds great promise as a contactless process able to rationally and rapidly manufacture complex nanostructures bringing innovative surface functions. The most critical challenge in controlling the growth of laser‐induced structures below the light diffraction limit is the absence of external order associated to the inherent local interaction due to the self‐organizing nature of the phenomenon. Here high aspect‐ratio nanopatterns driven by near‐field surface coupling and architectured by timely‐controlled polarization pulse shaping are reported. Electromagnetic coupled with hydrodynamic simulations reveal why this unique optical manipulation allows peaks generation by inhomogeneous local absorption sustained by nanoscale convection. The obtained high aspect‐ratio surface nanotopography is expected to prevent bacterial proliferation, and have great potential for catalysis, vacuum to deep UV photonics and sensing.

## Graphical abstract

A uniform formation of unconventional self‐organized patterns on the 100 nm scale has been generated by focused ultrafast light. Irradiated surface turns to a forest of self‐organized nanopeaks with a remarkable aspect ratio at the nanoscale. Laser beam polarization is timely controlled to prevent anisotropic energy absorption and to synchronize the laser energy delivery rate with the material dynamic response.

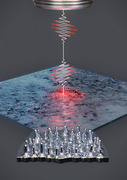

## 1 Introduction

Ordinary surfaces behave in extraordinary ways when tailored structures are designed, resulting in completely new physico‐chemical properties. New behaviors emerging from nano‐objects are steadily unraveled, generally attributed to quantum effects in condensed matter on mesoscopic scales.^[^ ^1^, ^2^, ^3^ ^]^ As well, when dealing with a large concentration of interacting nano‐objects, properties differing from the bulk counterparts result from their collective response.^[^ ^4^ ^]^ Different material properties can thus be defined by structuring at this scale, designing new thermal, mechanical, or optical characteristics. Multiscale and hierarchical features offer multifunctional surfaces able to imitate natural surface behaviors. Biomimetism examples relying on hierarchical high‐aspect‐ratio topographies include extreme hydrophobicity of lotus leaf surfaces,^[^ ^5^ ^]^ the synthesis of high‐density nanopillars to reproduce high adhesive forces of gecko feet,^[^ ^6^ ^]^ broadband antireflective coating relying on protuberances inspired by moth eyes,^[^ ^7^ ^]^ bactericidal spiky surface of cicada wings.^[^ ^8^ ^]^ In the latter case, mechano‐bactericidal nanostructures inflict critical membrane damage to microorganisms perched upon them, leading to subsequent cell lysis and death. Lethal mechanical forces causing bacterial cell death requires sharp and high aspect ratio geometries.^[^ ^9^, ^10^ ^]^ Similarly, black silicon, a nanomaterial synthetized under laser irradiation that exhibits high aspect ratio nanoprotrusions, has early been proposed to assist photovoltaic cells for better conversion efficiency but also for bactericidal activity.^[^ ^11^, ^12^ ^]^ Ultrashort laser is then a promising candidate to control both surface hydrophobicity and bacterial activity on a cellular level due to nanopeaks distinct aspect ratio, controllable concentration, and periodicity.^[^ ^13^, ^14^, ^15^, ^16^, ^17^ ^]^

For generating nanostructures, electron beam lithography,^[^ ^18^ ^]^ metal assisted chemical etching,^[^ ^19^ ^]^ or focused ion beam milling^[^ ^20^ ^]^ are routinely employed. However, these approaches do not allow for high‐throughput creation of topographic patterns on large‐scale surfaces. Nanoimprint lithography, an efficient replication method, is also able to address a wide range of pattern sizes and shapes but consists of a multi‐step process sequence.^[^ ^21^ ^]^ Ultrafast light processing greatly simplifies the design and fabrication process, becoming a practical route to fabricate user‐defined complex surface patterns with utmost precision reaching as well the 100 nm scale.^[^ ^4^, ^22^, ^23^ ^]^ In particular, self‐organized structures emergence observed below the ablation process strongly limit the thermal diffusion energy whereas the absorbed energy stays confined well into the nanoscale.^[^ ^23^ ^]^ Benefiting from their high concentration, replicability, and controllable periodicity and orientation, laser‐induced surface structures (LIPSS) can enable unique surface functionalization toward the control of tribological,^[^ ^24^ ^]^ optical,^[^ ^25^ ^]^ chemical, and biological^[^ ^26^ ^]^ surface properties on a nanometric scale. Squeezing light into tiny, subwavelength‐scale spaces results in self‐assembly of structures able to bypass the barrier of the light diffraction limit, in the µm scale, for large scale manufacturing. Near‐field nanostructuring associated with self‐organized patterns induced by ultrafast laser irradiation can reach lateral dimensions in the tens of nanometer and an amplitude defined by the thermal gradients. Self‐assembly of arrays of nanocavities of 20 nm diameter with a periodicity down to 60 nm has been realized with an unprecedented uniformity on extreme scales and the potentiality to access to large scales.^[^ ^27^, ^28^ ^]^ This confined optical response is critically dependent on the physical processes induced by light that can be guided in time and space.

Here we report on the synthesis of concentrated surface nanopeaks with a height up to ten times larger than their width on the nanoscale. To optimize this exceptional aspect ratio structures in terms of uniformity, yield, and scale, an optimal control approach is proposed by a synchronization of the laser energy delivery rate with the material dynamic response, upgrading the energy coupling. Crossed‐polarized ultrafast laser pulses enable to bring the surface far‐from equilibrium, relaxing into a liquid exhibiting natural convection currents. Upon multipulse irradiation, convective cells frozen by resolidification serve as precursors for the growth of inter‐cell regions spared from local field enhancement. This leads to a significant upgrade in laser processing, beyond the current state of the art, moving to an unexplored scientific field where light coupling and hydrodynamics flow act coherently and synergistically at the nanoscale to self‐organized metastructures.

## 2 Results

### 2.1 Surface Nanoengineering

**Figure 1:**
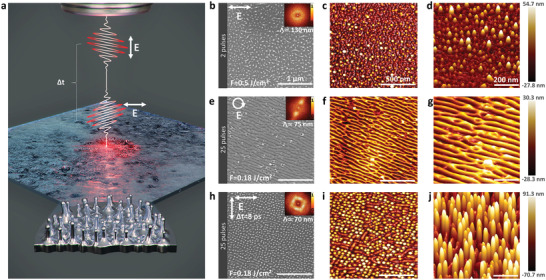
a) Schematic illustration of nanopeaks formation by femtosecond laser double pulse. b,e,h) Scanning electron microscopy with 2D Fourier Transform (FT). c,f,i) 2D atomic force microscopy. d,g,j) 3D atomic force microscopy of various surface morphologies of Ni(001) crystal irradiated with different laser parameters and polarizations. The number of laser shots is 2 single pulses (b), 25 single pulses (e), and 25 double pulses (h). The laser polarization *E* is indicated by the white arrow.

A (001) surface‐oriented nickel surface was photoexcited by 70 fs laser pulses at the central wavelength of 800 nm controlling laser peak fluence, number of double‐pulses sequences (*N*_DPS_) and polarization angle of single and double laser pulses. In the focal region, the laser‐induced nanopatterns exhibit remarkable topographical complexity and consistency depending on the beam features as revealed by scanning electron microscopy (SEM) and atomic force microscopy (AFM) and shown in **Figure** **1** using three different polarization strategies. Uniform micro‐spikes are usually obtained using a linearly polarized field near the ablation threshold at the micrometer scale with a high number of pulses to enhance positive feedback,^[^ ^29^ ^]^ while nonuniform nanospikes are obtained with a few number of pulses at high fluence to prevent LIPSS.^[^ ^30^ ^]^ These two approaches do not allow to produce homogeneous spiky structures at the nanoscale. To provide a benchmark to compare the nanostructure features, horizontal polarization irradiations were performed for 1 and 2 pulses at a peak laser fluence between 0.2 and 2 J cm^−2^. A low value of *N*_DPS_ were operated to prevent LIPSS formation. The optimal pattern of nanostructures were obtained for a single state of polarization at a laser peak fluence of 0.5 J cm^−2^ as presented in the SEM image of Figure 1b. The 2D FT does not display any particular symmetry of these unorganized nanostructures. Although chaotic, a maximal nanostructures height of 55 nm has been estimated by the AFM analysis as shown in Figure 1c,d.

Driven by near‐field light enhancement on local surface nanoreliefs, organized patterns were recently discovered with an ultimate scales down to tens of nanometers such as nanopeaks, nanobumps, nanohumps, and nanocavities at *N*_DPS_ = 25.^[^ ^27^, ^28^ ^]^ In order to avoid the formation of LIPSS, circular and cross‐polarized double pulses were applied to break the surface isotropy by controlling the laser peak fluence and the time delay between the cross polarized pulses. The formation of analogous nanostructures was reported by using circular polarization on tungsten metal.^[^ ^31^ ^]^ Consequently, Ni(001) surface irradiation has been performed with a circular polarization at a fixed *N*_DPS_ = 25 and a laser fluence between 0.1 and 0.24 J cm^−2^. Nanopeaks nanostructures have not been observed using circular polarization, high spatial frequency LIPSS (HSFL) patterns are observed at 0.18 J cm^−2^. Their periodicity Λ ≈ 75 nm was measured on SEM micrograph and confirmed with the 2D FT. The observed HSFL have a diagonal direction of ≈45° as shown in Figure 1e. Their height has been estimated by 2D and 3D AFM analysis of ≈30 nm as presented in Figure 1f,g, which are identical to the observed ones on Cr.^[^ ^32^ ^]^

Spontaneous and well organized nanostructures, named as nanopeaks^[^ ^28^ ^]^ are discovered at a specific laser fluence of 0.18 J cm^−2^ and a time delay of 8 ps and presented by the schematic illustration in Figure 1a. The performed 2D FT displays a diagonal symmetry of the observed nanostructures with a periodicity Λ ≈ 70 nm as observed in Figure 1h. The revealed nanopeaks have a high concentration, good organization, and large maximal height of ≈92 nm as observed in the 2D and 3D AFM images in Figure 1i, j. Thus, this demonstrates that double pulse cross‐polarization is a remarkably efficient strategy to boost the formation of high aspect ratio nanopeaks, able to functionalize uniformly the surface.

**Figure 2:**
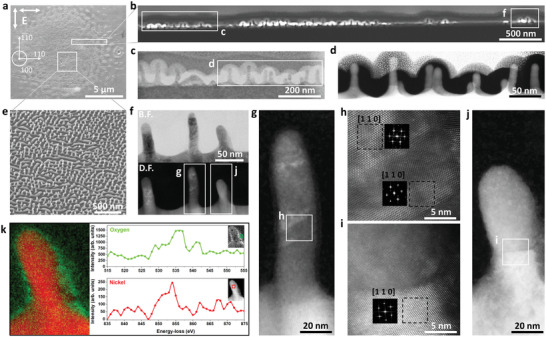
a) Scanning electron microscopy of a Ni(001) crystal irradiated by 25 double pulses at an incident laser fluence of 0.18 J cm^−2^ and a time delay of 8 ps between the two crossed polarizations. Crystallographic axes are indicated. b–d) A series of scanning transmission electron microscopy (STEM) images from the highlighted lamella in (a), extracted by a site specific method using a focused ion beam. These cross‐sectional views present the formation of nanopeaks and confirm their periodicity and their unique aspect ratio on a large surface. e) Tilted SEM image of the irradiated zone in (a). f–j) HR‐STEM images of the marked area in (b), presenting the oriented and disoriented crystals of the formed nanopeaks. The presented Ni(110) orientation of the cross sectional plane corresponds to the initial Ni(001) orientation of the top plane. k) STEM‐ADF image of a single nanopeak, acquired simultaneously with electron energy loss spectroscopy (EELS) spectral data in a spectrum image. O K‐edge and Ni L2‐edge are presented in the green and red charts.

The top‐view of the central area at *N*_DPS_ = 25 is shown in the SEM image as presented in **Figure** **2**a. A series of transmission scanning electron microscopy images from the highlighted lamellas in Figure 2a are presented in Figure 2b–d, displaying surface filled of boosted spontaneous nanopeaks with a diameter of ≈20 nm and elevation of ≈100 nm. Tilted SEM image of the irradiated zone is presented in Figure 2e showing a forest of nanopeaks. Nanopeaks periodicity in the scanning transmission electron microscopy (STEM) images confirms the periodicity calculated by the 2D FT in Figure 2b which is ≈70 nm. The layering of crystals with initial orientation and laser‐induced disorientions at the top of the peaks as presented in Figure 2g–j may result from very high cooling rates of up to 10^12^ K s^−1^, bringing the molten structure to a state of strong undercooling below the equilibrium melting temperature.^[^ ^33^ ^]^ The structural transformations could be created either by crystal twinning,^[^ ^34^ ^]^ stress‐induced dislocations,^[^ ^35^ ^]^ or by inhomogeneous epitaxial regrowth.^[^ ^33^ ^]^ Electron energy loss spectroscopy (EELS) entails the probing of core and valence level excitations caused by the laser absorption, where electrons are collected based on their energy after interacting with the specimen. This reveals electronic structure and bonding information at very high spatial resolutions down to single‐atom levels. We have exploited EELS to investigate the extent of ultrafast laser‐induced chemical modifications, in particular the uneven catalytic properties of hot nickel with oxygen due to inhomogeneous temperature resulting from non‐uniform topography as presented in Figure 2k.

### 2.2 Multipulse Dynamics of Nanopeaks Generation

**Figure 3:**
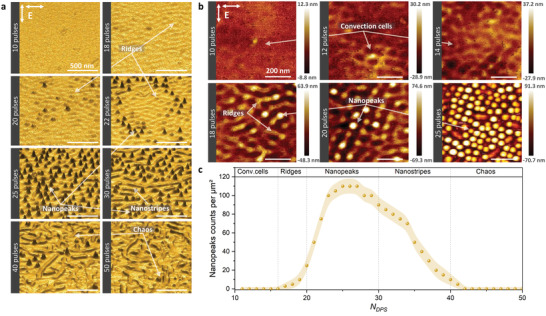
a) Pseudo‐3D scanning electron microscopy. b) 2D atomic force microscopy of a Ni(001) crystal irradiated by different pulses between 10 and 50 pulses at an incident laser fluence of 0.18 J cm^−2^ and a time delay of 8 ps between both laser crossed polarizations. Pulse‐to‐pulse dynamics of the nanopeaks formation by displaying the transformations from convection cells to ridges, nanopeaks, nanostripes, and chaos (a,b). c) Nanopeaks concentration evolution with the increasing *N*_DPS_ that underlines the role of laser dose to optimally synthesize a specific pattern.

The nanopeaks revealed in Figure 1h–j have an extraordinary potential in the application field. Understanding the physical mechanisms leading to the creation of these revealed nanostructures is vital. Mechanisms comprehension has a key role in promoting the manufacturing process and enhancing the control of nanopeaks shape and concentration, which will certainly make them compatible and adaptable for different function. As a result, a pulse‐to‐pulse growth dynamics investigation has been carried out by setting laser peak fluence to 0.18 J cm^−2^, time delay to 8 ps and pulse duration to 70 fs, varying the *N*_DPS_ from 10 to 50 as presented in **Figure** **3**. The impacts are independent as performed on a different surface location at a fixed pulse value. They were observed ex situ to progressively unveil the activation, triggering, and development of nanopeaks.

A pseudo 3D reconstruction of the SEM images was performed for Figure 3a, visibly presenting the transitions from a smooth initial surface to the formation of ridges, nanopeaks, nanostripes, and chaos for different *N*_DPS_. The AFM images displays the nanopeaks formation at a higher magnitude, covering the missing height information of the 3D reconstructed SEM images, and presenting the formation of convection cells,^[^ ^36^ ^]^ ridges, and nanopeaks for different *N*_DPS_ as shown in Figure 3b. To summarize, the nanopeaks counts per µm^2^ versus *N*_DPS_ chart displays the nanopeaks generation and concentration for different *N*_DPS_ as presented in Figure 3c.

At *N*_DPS_ < 10, the irradiated surface topography looks identical to the initial surface topography before laser irradiation. Differently, at 10 ⩽ *N*_DPS_ ⩽ 14 , convection cells appear on the surface with a diameter of ≃ 70 nm and a maximum height of ≃ 37 nm at N_DPS_= 14 as presented in Figure 3b. Afterward, the ridges started to form on the convection cells at 18 ⩽ *N*_DPS_ ⩽ 20. Subsequently, at 20 ⩽ *N*_DPS_ ⩽ 30, the nanopeaks started to appear and grow from a height of ≃ 64 nm to reach a maximum height of ≃ 92 nm at *N*_DPS_ = 25. For this dose regime, the nanopeaks concentration reaches its optimal condition with a maximum count of 110 per µm^2^. The nanopeaks concentration decreases progressively while increasing the *N*_DPS_ > 28 and the nanopeaks are replaced by nanostripes at 30 ⩽ *N*_DPS_ ⩽ 40. Chaotic nanostructures started to appear on the surface. Eventually, they are formed for higher *N*_DPS_.

### 2.3 Nanopeaks Generation and Growth Mechanism

**Figure 4:**
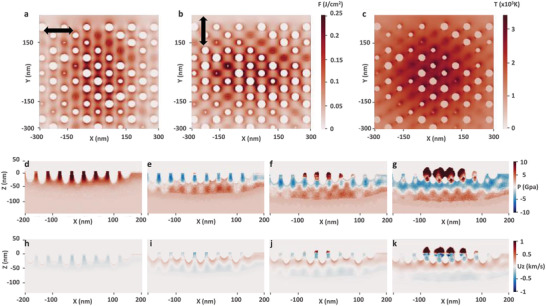
a,b) Energy density absorbed below Ni surface with hexagonal distribution of nanocavities by the first (a) and the second cross‐polarized pulse (b). c) Equilibrium temperature distribution after double pulses applied with a delay of 8 ps. Distributions are shown in the plane perpendicular to laser incidence (along *Z*) 10 nm below the upper surface level *Z* = 0. d–g) Instantaneous EOS pressure *P* and h–k) fluid velocities *U*_*z*_ beneath the surface 4 ps (d,h), 8 ps (e,i), 16 ps (f,j), and 24 ps (g,k) after the first pulse excitation. The cross‐sections are taken for *Y* = 0 in the propagation plane.

Early in the feedback process, near‐field enhancement on local depression turns to nanocavities that self‐organize into an hexagonal 2D lattice. This results from hydrothermal flows guided by surface tension and rarefaction forces developing a thermoconvective instability at the nanoscale and, similarly to well‐known Rayleigh–Bénard–Marangoni instabilities, generate convective cells as shown in Figure 3b.^[^ ^27^, ^36^ ^]^ The growth of the laser‐induced structures from quasi‐hexagonal cavities to uniform nanopeaks is analyzed by combined electromagnetic and hydrodynamic approaches explained in numerical details section. The absorbed energy deposition on the Ni surface, nanostructured with some hexagonally arranged nanocavities, is first calculated by 3D Maxwell equations and shown in **Figure** **4**a,b for cross‐polarized ultrashort laser pulses. For the nanocavity concentrations observed in the experiments (sub‐wavelength inter‐distances of ≈80 nm), strong collective effects in quasi‐periodic lattices are expected, deviating from the individual response of the nanocavities. For the sake of generality, nanocavity distributions with randomly varying depths and slight deviations of the centers from the exact hexagonal order were considered, in accordance to the experimental observations done in Figure 3b. These small imperfections were shown to have negligible effects on the qualitative optical response of the quasi‐periodic system. Nevertheless, the ratio between the intercavity distance and the cavity radius should be small enough (<5) to support strong near‐field enhancement, induced by neighbor nanocavities (see Supporting Information for details).

The distance between the closest nanocavities positioned perpendicular to laser polarization defines the dominating regime for each laser pulse. For instance, the intensity averaged over the period is amplified along the lines perpendicular to the polarization in Figure 4a if the distance is small enough. The enhancement along the diagonal axes is also present, though a weaker effect. In contrast, the intensity is amplified dominantly along the diagonal axis when the inter‐distance along the horizontal axis is wider in Figure 4b. Furthermore, the application of cross‐polarized pulses results in the uniform total absorbed energy deposition (and therefore, temperature distribution) in the vicinity of nanocavities, cancelling the paramount directional absorption from individual near‐field effects. As a result, the combination of double pulses renders higher temperatures along one of the diagonal axis of the hexagonal cavities as shown in Figure 4c rather than perpendicular to one of the applied fields. The equilibrium temperatures along the diagonal axis are high enough to induce phase transitions, that is melting of the Ni surface. Their consequence might be seen in the experimental results prior to the formation of nanopeaks.

In the propagation plane, the combined action of cross‐polarized pulses results in the increased temperatures on the top of the nanostructures. This is the consequence of the cancelling of directional individual responses of the nanocavities, which would amplify the intensity beneath the nanocavities if a single perpendicularly polarized pulse is applied. The fluid dynamics simulations are further performed in order to elucidate the driving mechanisms of the nanopeak growth.

The instantaneous pressure *P* and fluid velocities *U*_*z*_ are provided in Figure 4d–k. The snapshots are taken at different stages of the structure evolution: heating by the first pulse (d,h), the surface state at the moment when the second pulse is applied (e,i), temperature equilibrium attained after heating by the second pulse (f,j), and, finally, the growth and saturation of the nanopeaks (g,k). The positive velocities indicate the potential material flow above the surface, whereas the negative values stand for the movement below the surface, for instance, shock wave propagation in the bulk of the material. The role of the delay between the cross‐polarized pulses (here, 8 ps) becomes evident while comparing the pressure distributions (d) at first and (e) at second pulse energy deposition. The negative pressures of −3 GPa indicate the initiated melting processes at the top of the nanostructures, therefore, the second pulse starts heating the peaks in the liquid phase. The difference between ultrafast heating of solid and liquid Ni with a reduced density originates from the instantaneous drop in temperature keeping the density unchanged and elevated transition temperature (from positive to negative pressures *P*(ρ, *T*_*i*_)) for a liquid state. As a result, the positive pressures (compressive stress of ≈8 GPa) can be built at the top of the nanostructures, where the difference between negative pressures below the surface and on the top creates upwards fluid movement above the initial level of surface. The peaks can grow by tens of nanometers according to the numerical predictions as shown in Figure 4g and are latter stopped by the dominating diffusion processes. This localized high mechanical stress is probably the cause of the crystal disorientation revealed by HR‐STEM in in Figure 2g–j.

The experimental results for different number of applied pulses (20–30) in Figure 3a indicate that the nanopeaks grow swiftly up to ≈100 nm heights and then saturate, covering the irradiated area almost homogeneously. The growth saturation can be explained by drastic changes in the optical response of the surface topography, resulting in a smaller field enhancement on the top of the nanostructures (see Supporting Information for the electromagnetic and multipulse simulations of the surface topography evolution). As a result, the temperature increase drops down and the pressure gradients are not strong enough to contribute to further growth of the nanopeaks above the established height. Instead, the energy is more efficiently absorbed on the surface, resulting in less regular long nanostripes, connecting the neighbor nanopeaks for a higher number of applied pulses (30–50).

The experimental results and the performed calculations allow us to get insight into the nanostructure formation mechanisms as well as into the specific laser irradiation conditions required to control over the observed phenomenon. The unique role of cross‐polarized laser pulses with equal amount of energy in concentrating light and heat at the nanoscale and in avoiding the formation of field‐aligned patterns has been already evidenced in previous works.^[^ ^27^, ^28^ ^]^ Specifically, the nanocavities and the nanobumps were attributed to convection instability in a thin liquid layer of Ni. As soon as these nanostructures densely and homogeneously cover the surface after multiple pulse irradiation, the light is trapped by the sub‐wavelength structures and results in even more extreme conditions in a thin liquid layer. Additional parameter which is crucial is a choice of double pulse delay. If a delay is much shorter than the time required for energy transfer between electrons and lattice, both pulses interact with solid material and their action sums up. If a delay is much longer than the electron‐ion transfer time, the diffusion effects tend to produce homogeneous temperature and pressure distributions, with less pronounced nanoscale hot spots. The intermediate regime is of particular interest because the temporal scales correspond to the initiation of phase transitions. Additionally, the fluence should be adapted in order to induce pronounced material modifications only where the light is confined by the nanostructures to avoid the uncontrolled damage. The influence of pulse duration in sub‐picosecond range indicates the potential role of transient optical properties out of thermal equilibrium as well as the liquid state gradients in the efficiency of nanoscale energy deposition.

### 2.4 Nanopeaks Aspect Ratio and Distribution Control

**Figure 5:**
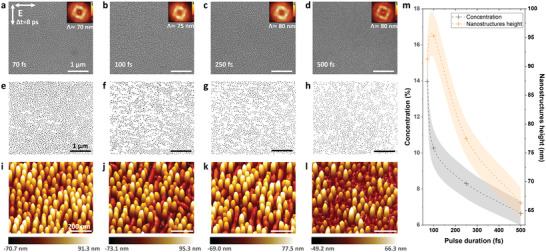
a‐d) Scanning electron microscopy with 2D FT. e–h) Concentration interpretation of scanning electron microscopy. i–l) 3D atomic force microscopy images of a Ni(001) crystal irradiated by 25 double pulses at an incident laser fluence of 0.18 J cm^−2^, time delay of 8 ps between the two crossed polarizations and pulse duration between 70 and 500 fs. m) The chart presents the crucial role of pulse duration in controlling the concentration and height of the formed nanopeaks.

Controlling nanopeaks aspect ratio and concentrations will definitely make them compatible with several types of applications. The essential role of laser parameters in regulating nanopeaks shape and concentration for a constant laser peak fluence of 0.18 J cm^−2^, a time delay of 8 ps, and a *N*_DPS_ of 25 as presented in **Figure** **5**, by varying the pulse duration between 70 and 500 fs progressively. The nanopeaks periodicity has slightly increased from 70 to 80 nm while increasing the pulse duration from to 70 to 500 fs as presented by the 2D FT of the Figure 5a–d.

The nanostructures concentration was extracted from the SEM images, as shown on Figure 5e–h and synthetized in Figure 5m. The concentration has decreased from 14% to 6.5% while increasing the laser pulse duration from 70 to 500 fs accordingly, in agreement with the increase in the period of the 2D FT interpretation. The nanopeaks height was determined by an AFM analysis presented in Figure 5i,j and averaged for each conditions in Figure 5m. Nanopeaks height has decreased of about 30% from a maximum height of 95 nm at 100 fs to 66 nm at 500 fs. This suggests that the mechanical momentum defined by pulse duration plays a crucial role in controlling the aspect ratio, the periodicity as well as the concentration of the formed nanopeaks.

## 3 Conclusion

We conclude by pointing out the proposed nanostructuring strategy, relying on a timely‐controlled polarization feedback, opens the route for producing uniform high‐aspect ratio structures for nanoengineering metamaterials. A flat surface turns into a forest of nanopeaks with a remarkably high aspect‐ratio (5:1) and a sub‐100 nm periodicity. This nanopatterning could have a great impact in surface bioengineering as the size, shape, and distance of the nanopeaks can prevent virus activation and determine the ability of cells to adhesion, proliferation, and differentiation, establishing bactericidal performances.^[^ ^10^, ^37^ ^]^ Engineering of high aspect ratio regular and densely packed nanostructures opens also broad optical opportunities toward phase and polarization state manipulation, metaphotonics with plasmonic metastructures and extreme light confinement at the nanoscale, strongly enhancing the surface nonlinear optical response in deep and vacuum UV spectral regions.^[^ ^38^, ^39^ ^]^ Ultrafast laser process offers unprecedented control for manufacturing appropriate artificial media with nanofeatures surpassing those of any naturally‐occurring surfaces with expected innovative applications in biomedecine, nanocatalysis, and metaphotonics.

## 4 Experimental Section

### Materials and Laser Irradiation

A monocrystal oriented in (001) direction had been irradiated by femtosecond laser pulses at different number of pulses, laser fluences, and pulse durations. Ni single crystals were preferred with an initial arithmetic mean surface roughness *R*_a_ < 5 nm to insure the uniformity of the nanopeaks formation. Initial surfaces were prepared by conventional metallographic procedures. The nanopeaks on the surfaces of Ni(001) targets were produced using a Ti: Sapphire laser from Coherent (Legend Elite Series) powered by an integrated revolution pump laser and seeded by a Viatara‐S‐Oscillator. The laser had a central wavelength of 800 nm and a repetition rate of 1 kHz with a controllable pulse duration in the femtosecond range. A half‐wave plate was installed in one interferometer path to optionally rotate the polarization direction. A Mach–Zehnder interferometer was used to divide the incoming laser beam into two beams and to combine the effect of crossed‐polarization with controllable inter‐pulse delay of a resolution better than 0.15 ps. Detailed description of the laser setup could be found in our previous paper. The linear, circular, and cross‐polarization pulses were attenuated and focused through a 250 mm focal length at a normal incidence angle. The focused laser spot exhibited a Gaussian profile and the spot size (at 1/e^2^) measured 2ω_0_ = 56 µm determined by the D^2^ method.

### Microscopy

The analysis of surface morphology of the laser irradiated area was performed using scanning electron microscopy (Nova NanoSEM), equipped with a field emission gun. Atomic force microscopy (Bruker Dimension ICON) was used to characterize the surface morphology in 2D and 3D in tapping mode using a supersharp OPUS tip (240AC‐SG), gold coated to ensure high and stable laser reflectivity with a diamond‐like spike designed for high resolution AC mode AFM imaging of soft samples and a resolution <1nm. Topological interpretations were performed using NanoScope Analysis software. Microstructural examination of the cross sectional cut was performed using transmission electron microscopy. High‐resolution transmission electron microscopy (HR‐STEM) was performed using a JEOL NEOARM microscope, equipped with a spherical aberration corrector, operating at an acceleration voltage of 200 kV equipped with STEM‐ADF and EELS. TEM lamella preparation was carried out using a FIB/SEM workstation (NVision 40; Carl Zeiss Microscopy GmbH) combining a SIINT zeta FIB column (Seiko Instruments Inc. NanoTechnology) with a Gemini I column. The NVision 40 platform was equipped with a multinozzle SIINT gas injection system (GIS). Careful milling and low kV ion polishing were applied to obtain the final TEM lamellae (thickness around 100 nm). Precautions were taken during the preparation to minimize the curtain effect and surface implantation.

### Statistical Analysis

The used function to count the number of items or observations was based on ref. [40]. This method examined in input an 8 −bit image considering the values 0 for the background and 255 for the observation. The input image was then converted into a labeled image. The pixels values of the output image were between 0 and *N* − 1, where *N* is the number of items (nanopeaks here), by associating input pixels equal to 255 spatially connected owing to decision trees. The analyzed size was 5× 5µm^2^ and the error bars were represented by B‐Spline and Bezier methods for Figures 3c and 5m, respectively.

### Numerical Details

3D simulations of the energy deposition and material modification on the Ni surface were performed. The absorbed energy density was calculated based on the solution of Maxwell equations (for electric and magnetic fields $\vec{E}$ and $\vec{H}$) by using the finite‐difference time‐domain (FDTD) method with the auxiliary equation for polarization current $\vec{J}$ (Drude model for Ni metal with ω_pl_ and ν plasma and collision frequencies)^[^ ^41^ ^]^ as follows

(1) $$\begin{array}{c} \left\{\begin{array}{c} \frac{\partial \vec{E}}{\partial t}=\frac{\nabla \times \vec{H}}{\varepsilon_{0}}-\frac{1}{\varepsilon_{0}}\vec{J}\\ \frac{\partial \vec{H}}{\partial t}=-\frac{\nabla \times \vec{E}}{\mu_{0}}\\ \frac{\partial \vec{J}}{\partial t}+\vec{J}\nu =\varepsilon_{0}\omega_{pl}^{2}\vec{E} \end{array}\right. \end{array}$$

The absorbed energy was defined as *I*α_abs_, where $I=\frac{1}{2}\sqrt{\frac{\varepsilon_{0}}{\mu_{0}}}{\left|\vec{E}\right|}^{2}$ is the intensity and α_abs_ is the absorption coefficient related to the extinction coefficient *k* as α_abs_ = 4π*k* /λ. Then, a two‐temperature model (TTM) was implemented to resolve electron‐ion heat transfer and diffusion^[^ ^42^ ^]^ and compressible Navier–Stokes equations^[^ ^36^, ^43^ ^]^ were applied as follows

(2) $$\begin{array}{c} \left\{\begin{array}{c} C_{e}\frac{\partial T_{e}}{\partial t}=\nabla \cdot \left(k_{e}\nabla T_{e}\right)-\gamma_{\text{ei}}\left(T_{e}-T_{i}\right)+I\alpha_{\text{abs}}\\ \rho C\left[\frac{\partial T_{i}}{\partial t}+\vec{u}\cdot \nabla T_{i}\right]=\nabla \left(k_{i}\nabla T_{i}\right)+\gamma_{\text{ei}}\left(T_{e}-T_{i}\right)\\ \frac{\partial (\rho \vec{u})}{\partial t}+\left(\vec{u}\cdot \nabla \right)\left(\rho \vec{u}\right)+\left(\rho \vec{u}\right)\nabla \cdot \vec{u}\\ \;=-\nabla \left(P_{e}+P_{i}\right)+\mu \nabla^{2}\vec{u}+\frac{1}{3}\mu \nabla \left(\nabla \cdot \vec{u}\right)\\ \frac{\partial \rho }{\partial t}+\nabla \cdot \left(\rho \vec{u}\right)=0, \end{array}\right. \end{array}$$

where $\vec{u}$ and ρ are the fluid velocity and density, *T*_e_ and *T*_i_ are the electron and ion temperatures, *P*_e_, *C*_e_, and *k*_e_ are the electronic pressure, the electron heat capacity and conductivity evaluated based on the results of ab initio calculations,^[^ ^44^ ^]^ *P*_i_ is the lattice pressure defined by the equation of state (EOS),^[^ ^45^ ^]^ µ, *C*_i_, and *k*_i_ are the ion viscosity, heat capacity, and thermal conduction.

Hexagonal distribution of nanocavities (half‐spheres of radii *R* ± Δ*R* = 30 ± 15 nm with centers on *Z* = 0 and inter‐distances of *L* ± Δ*L* = 80 ± 10 nm) was initially set on the Ni surface. The cross‐polarized pulses with combined fluence of *F* = 0.18 J cm^−2^ were applied with a delay of 8 ps as in experimental irradiation conditions.

## Conflict of Interest

The authors declare no conflict of interest.

## Supporting information

Supporting Information

## Data Availability

The data that support the findings of this study are available from the corresponding author upon reasonable request.
