# Supplementary material for: Boosted Spontaneous Formation of High‐Aspect Ratio Nanopeaks on Ultrafast Laser‐Irradiated Ni Surface
Source: Adv Sci (Weinh). 2022 May 26;9(21):2200761. doi: 10.1002/advs.202200761 (PMC9313481; doi:10.1002/advs.202200761)
Supplement: Supplementary file 1 — Supporting Information [file ADVS-9-2200761-s001.pdf]

ADVANCED SCIENCE
Open Access

Supporting Information

for *Adv. Sci.*, DOI 10.1002/advs.202200761

Boosted Spontaneous Formation of High-Aspect Ratio Nanopeaks on Ultrafast Laser-Irradiated Ni Surface

*Anthony Nakhoul, Anton Rudenko, Claire Maurice, Stéphanie Reynaud, Florence Garrelie, Florent Pigeon and Jean-Philippe Colombier**

Supplementary Materials for « Boosted Spontaneous Formation of High-Aspect Ratio Nanopeaks on Ultrafast Laser-Irradiated Ni Surface »

Anthony Nakhoul[1,3] | Anton Rudenko[2] | Claire Maurice[3] | Stéphanie Reynaud[1] | Florence Garrelie[1] | Florent Pigeon[1] | Jean-Philippe Colombier[1*]

1 | SUPPLEMENTARY MATERIALS

1.1 | Configuration of the nanocavities

The configuration (hexagonal periodic arrangement, period 80 nm) and the sizes of nanocavities (radius 30 nm) were taken close to the experimental results where the convection cells were pre-formed after irradiation by 12 − 14 consequent pulses in Figure 3b in the manuscript. These two values have been varied in simulations with standard deviations of 15 nm for radii and 10 nm for hole inter-distances. Such deviations do not influence the qualitative results. Nevertheless, the long separation between the nanocavities (> 5 radii of nanocavities) significantly decreases the intercoupling effects and the near-field enhancement (see Figure 1 for the electromagnetic simulations). In such configurations, not observed experimentally, each nanocavity exhibits mostly individual optical response to laser irradiation. Therefore, the nanopeaks are not expected to be formed in case of unproportionally small nanocavities or large distances between them.

1.2 | Near-field and far-field

In the configuration observed in the experiments and considered in the simulations, light is confined to the area of densely packed nanostructures (nanocavities/nanopeaks) and the near-field enhancement effects are limited to vicinity of these nanostructures and occur to be much stronger than the far-field enhancement effects. In laser processing, the appearance of LSFL (nanostructures with near-wavelength periodicity, low spatial frequency LIPSS) is commonly a sign of the dominant role of the far-field interferences. In the considered regime, laser pulse energy is weaker than required to produce modifications from the far-field patterns and, consequently, form LSFL structures in the irradiated area. The near-wavelength LIPSS were observed experimentally for a higher laser pulse fluence.

1.3 | Saturation in the nanopeak growth

The effects of nanostructure growth saturation commonly occur for most types of laser-induced periodic surface structures (LIPSS) upon multi-pulse femtosecond laser irradiation. The physical reasons for this saturation might have both electromagnetic and hydrody-

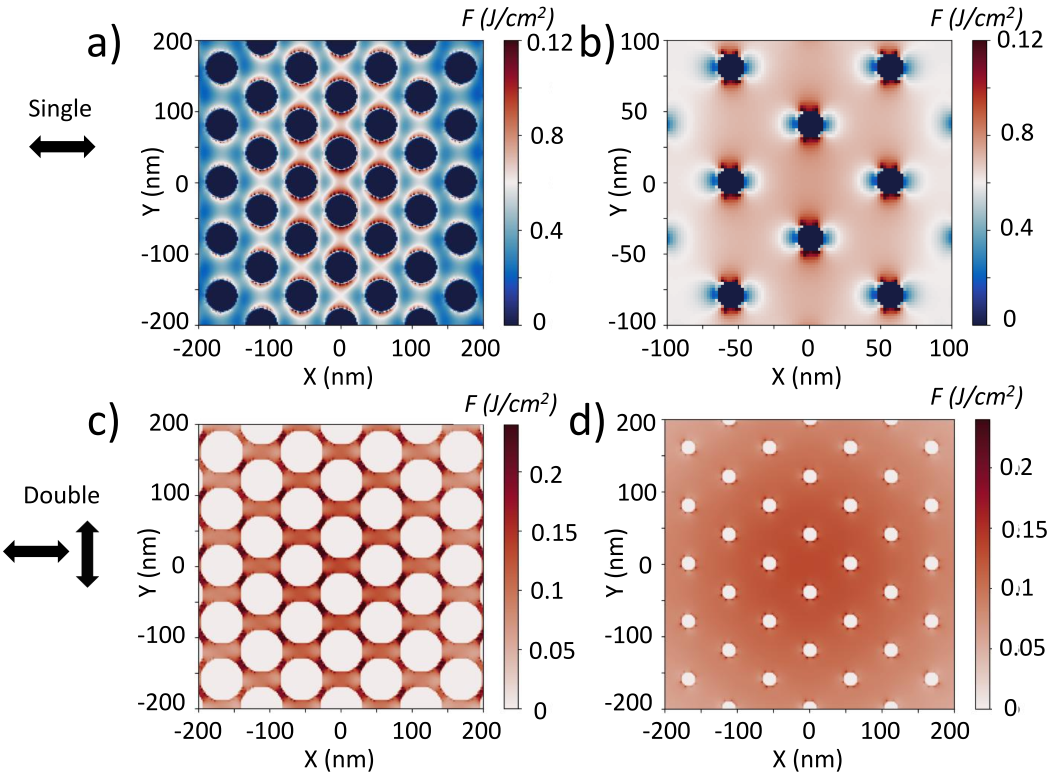

FIGURE 1 (a, b) Single (F = 0.09 J/cm^2) and (c, d) combined (F = 0.18 J/cm^2) optical response (fluence, absorbed energy) by two orthogonal double pulses in case of hexagonal nanocavity distributions with 80 nm distances between neighbor nanocavities and radii (a, c) R = 30 nm, (b, d) R = 10 nm. The results are presented in the transverse plane, perpendicular to laser propagation. For small cavities, there is no visible near-field interaction between separate nanocavities.

namic origins. The hydrodynamic origin in non-ablative regime (fluence is below the ablation threshold for material removal by phase explosion) was discussed for LIPSS structures in [Phys. Rev. B 99, 235412 (2019)], concluding that the sub-surface cavitation process in the tips of the nanostructures limits the growth due to strong cavity-induced electric field enhancement and higher surface temperatures after subsequent pulse irradiation. This scenario is not confirmed for thinner nanopeak structures, where the structural properties of individual nanopeak were investigated by several methods in Figure 2 in the manuscript and no cavitation has been observed. The electromagnetic scenario relates the growth saturation of the nanostructures to the decrease in the near-field enhancement upon the evolution of surface topography (growth of the nanopeaks) during multi-pulse femtosecond laser irradiation. To investigate this scenario, we have performed electromagnetic simulations for the absorbed energy distribution on Ni surface with the nanopeaks of different heights. Cross-polarized pulses would induce different intensity distributions; however, it is sufficient to consider one pulse in the chosen cross-section, with the intensity enhancement in the tip of the initial structure. The orthogonal pulse would in contrast enhance the field beneath the nanocavities, qualitatively independent of the

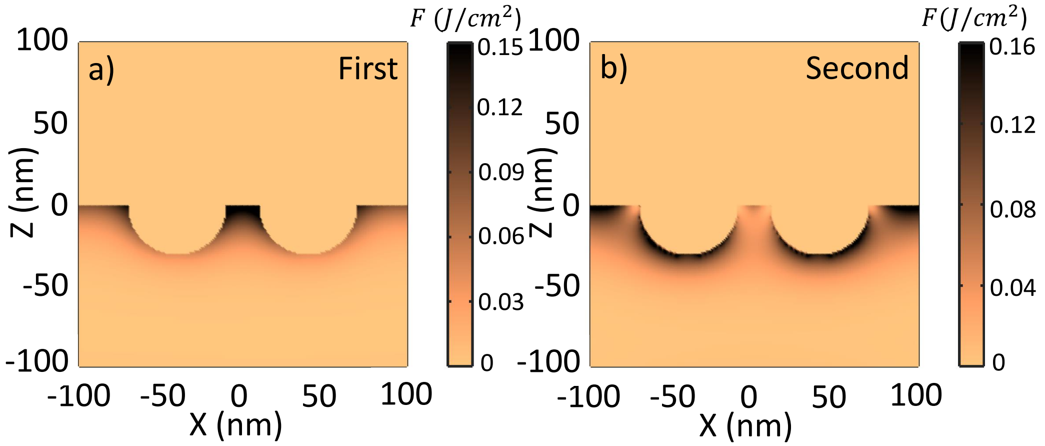

FIGURE 2 Absorbed energy (fluence) on Ni surface with a couple of nanocavities by two orthogonal laser pulses (first, second), each one with fluence of 0.09 J/cm^2 in the propagation cross-section. The field in enhanced in between the nanocavities for the first pulse, while beneath the nanocavities for the second.

nanopeak height (see Figure 2). For this reason, we focus our discussion on the first pulse (the results are shown in Figure 3).

With the increasing heights of nanopeaks (> 20 nm), the energy transferred to heat by the pulse would decrease. The field is reduced in the tip of the structures and, therefore, there are no pressure gradient forces that would stimulate the consequent growth of the nanopeaks by next pulses. This result is supported by the experimental results: the height of the nanopeaks saturated below 100 nm and no further growth was observed by applying higher number of pulses.

The evolution of the nanopeaks upon the action of the subsequent double cross-polarized laser pulses has been simulated. For each new pulse, the newly established surface topography with laser-induced nanostructures has been considered. Growth saturation of the nanopeaks has been observed for $N > 3$. The origin of this saturation is related to the reduced electromagnetic field inside the nanostructures exceeding a certain height, and, as a result, reduced temperatures and pressures. The snapshots in Figure 4 show the maximum instantaneous temperatures and pressures at the moment of the nanostructure growth (24 ps after the first pulse excitation) for different pulses ($N = 2$, $N = 3$, $N = 5$). As the structures grow, the maximum temperatures and pressure gradients decrease and do not contribute to a pronounced growth above the initial surface level. The height is almost unchanged for $N = 3$ and $N = 5$. This growth saturation might be in the origin of the homogeneity of the final nanopeak structures observed in the experiments.

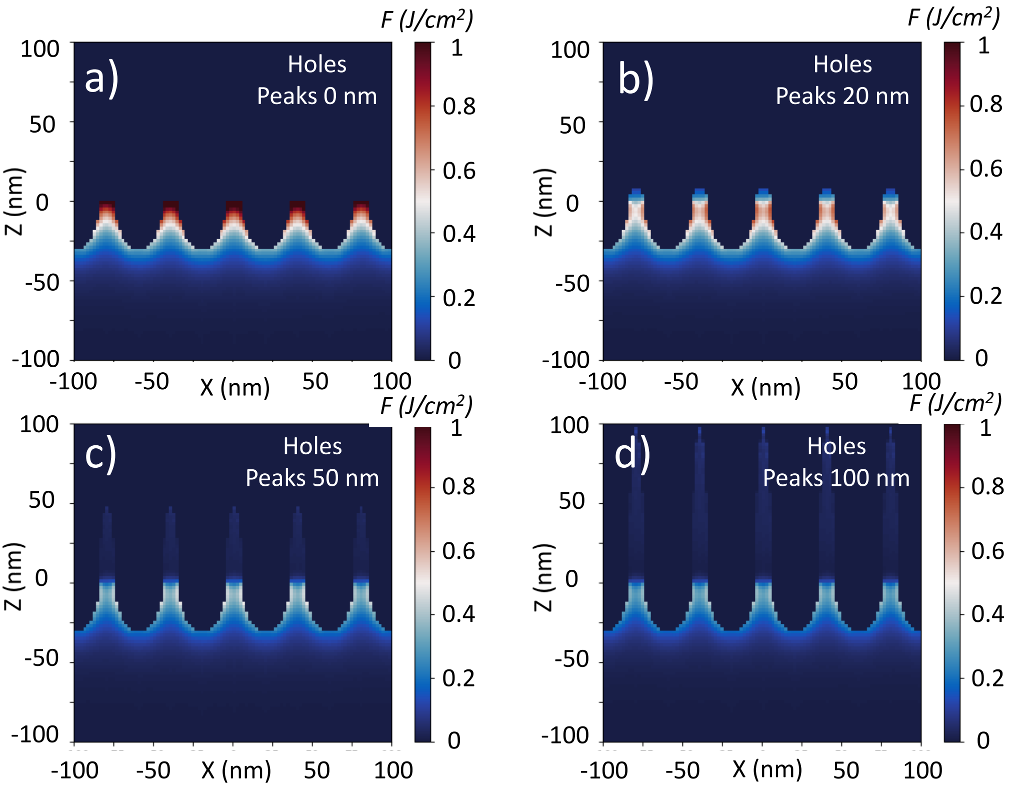

FIGURE 3 Fluence distribution on Ni surface with pristine nanocavities and nanopeak heights of (a) 0 nm, (b) 20 nm, (c) 50 nm, (d) 100 nm. Incidence fluence of 0.09 J/cm^2 (first pulse). The results are shown in the propagation cross-section.

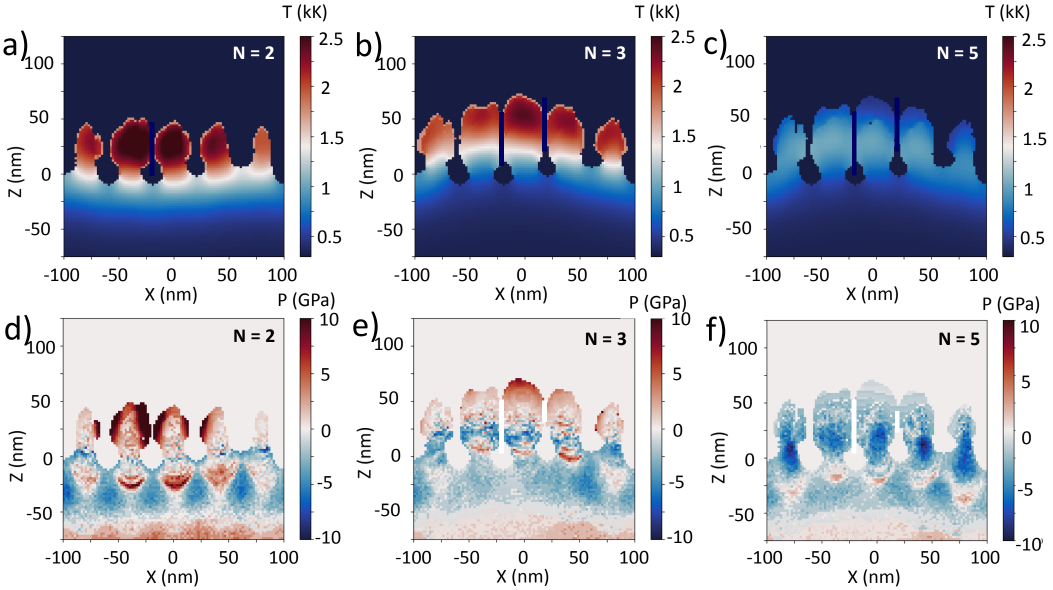

FIGURE 4 Temperature (a-c) and pressure (d-f) snapshots 24 ps after the first pulse excitation for different number of pulses (up to $N = 5$) upon the evolution of one surface morphology. The images show that the lower temperatures and pressures are attained in the laser-induced nanostructures after $3-5$ pulses and the heights of the peaks saturate. This saturation is further explained by electromagnetic simulations for the regular peaks of different heights with the results summarized in Figure 3.
